# Current and future therapeutic strategies for Alzheimer’s disease: an overview of drug development bottlenecks

**DOI:** 10.3389/fnagi.2023.1206572

**Published:** 2023-08-03

**Authors:** Yong Peng, Hong Jin, Ya-hui Xue, Quan Chen, Shun-yu Yao, Miao-qiao Du, Shu Liu

**Affiliations:** ^1^Neurology Department, The First Affiliated Hospital of Hunan Traditional Chinese Medical College, Zhuzhou, Hunan, China; ^2^Neurology Department, The Third Affiliated Hospital of Hunan University of Chinese Medicine, Zhuzhou, Hunan, China

**Keywords:** Alzheimer’s disease, β-amyloid protein, tau protein, mitochondria-targeting, multi-targets, clinical trials

## Abstract

Alzheimer’s disease (AD) is the most common chronic neurodegenerative disease worldwide. It causes cognitive dysfunction, such as aphasia and agnosia, and mental symptoms, such as behavioral abnormalities; all of which place a significant psychological and economic burden on the patients’ families. No specific drugs are currently available for the treatment of AD, and the current drugs for AD only delay disease onset and progression. The pathophysiological basis of AD involves abnormal deposition of beta-amyloid protein (Aβ), abnormal tau protein phosphorylation, decreased activity of acetylcholine content, glutamate toxicity, autophagy, inflammatory reactions, mitochondria-targeting, and multi-targets. The US Food and Drug Administration (FDA) has approved five drugs for clinical use: tacrine, donepezil, carbalatine, galantamine, memantine, and lecanemab. We have focused on the newer drugs that have undergone clinical trials, most of which have not been successful as a result of excessive clinical side effects or poor efficacy. Although aducanumab received rapid approval from the FDA on 7 June 2021, its long-term safety and tolerability require further monitoring and confirmation. In this literature review, we aimed to explore the possible pathophysiological mechanisms underlying the occurrence and development of AD. We focused on anti-Aβ and anti-tau drugs, mitochondria-targeting and multi-targets, commercially available drugs, bottlenecks encountered in drug development, and the possible targets and therapeutic strategies for future drug development. We hope to present new concepts and methods for future drug therapies for AD.

## 1. Introduction

Alzheimer’s disease (AD) is a chronic progressive disease with a hidden onset, unknown etiology, and long-term course. It is characterized mainly by cognitive dysfunction, such as aphasia and agnosia, and mental symptoms, such as hallucinations, delusions, and behavioral abnormalities, which significantly reduce the quality of life of older people (Alzheimer’s Association, 2022). The number of patients with AD worldwide is expected to exceed 150 million by 2050, according to the 2022 Alzheimer’s Disease Facts and Figures report (Man et al., 2023). Despite extensive research, the etiology of AD is complex, and its pathogenesis remains unknown. The primary hypotheses are abnormal deposition of beta-amyloid protein (Aβ), abnormal phosphorylation of tau protein, and nervous system inflammation, among others. Unfortunately, no drugs that can block AD progression are currently available.

Five drugs, Tacrine, Donepezil, Carbalatine, Galanthamine, and Memantine, have been approved by the Food and Drug Administration (FDA) for clinical use. Recently, FDA also approved Lecanemab. The first four types of drugs are acetylcholinesterase inhibitors (AChEIs), which can inhibit the activity of acetylcholinesterase (AChE) to prevent the degradation of acetylcholine in the synaptic gap, increasing the cholinergic effects, maintaining neuronal activity, and improving memory and learning abilities. Memantine is an N-methyl-D-aspartate (NMDA) receptor antagonist that can reduce the neurotoxicity of excitatory amino acids in the synaptic cleavage and reduce neuronal apoptosis. However, these drugs only manage symptoms and delay the onset of AD but do not cure it. Lecanemab (BAN2401), an IgG1 monoclonal antibody, was well tolerated during the trial, although some participants experienced ARIA-E (Swanson et al., 2021). In a multicenter, double-blind, 18-month Phase III trial, Lecanemab reduced amyloid markers in patients with early AD; longer trials are required to determine the effectiveness and safety of this drug (van Dyck et al., 2023).

Several drugs are undergoing clinical trials for AD, but unfortunately, many have been terminated because of poor efficacy or large adverse reactions. Existing clinical trials have focused on two pathological features of AD: amyloid plaques (Aβ), tau protein, mitochondria-targeting, and multi-targets. Therefore, we classified AD’s future treatment strategies into four main aspects: resistance against Aβ or anti tau protein treatment, mitochondria-targeting, and multi-targets agents.

## 2. Brief introduction of the physiological and pathological basis of AD

There were nine major mechanisms of the physiological and pathological basis of AD, such as Aβ deposition (Basisty et al., 2020), abnormal phosphorylation of tau protein (Jouanne et al., 2017; Hampel et al., 2019; Jo et al., 2020), decreasing acetylcholine activity (Cho et al., 2019; Babic Leko et al., 2021), glutamate toxicity (Ogbodo et al., 2022), autophagy (Reddy and Oliver, 2019; Luo et al., 2020), inflammatory response (Nho et al., 2019), neurovascular mechanism and mitochondrial hypothesis (Reddy and Reddy, 2017; Shevtsova et al., 2017, 2021; Wilkins and Morris, 2017; Liu et al., 2019), as well as “multi-target” agents (Makhaeva et al., 2019).

### 2.1. Aβ deposition

Beta-amyloid protein is an important biomarker used in the diagnosis of AD. Aβ plaques are formed by the hydrolysis of amyloid precursor protein (APP) through α, β, and γ secretory enzymes. Specifically, a portion of APP is cleaved by β-site APP-cleaving enzyme-1 (BACE-1) to produce a membrane-bound carbon terminal 99 amino acid fragment known as C99. C99 is then cleaved by γ-secretase to form Aβ1-40 and Aβ42. While Aβ monomers are typically soluble in small amounts and have no neurotoxicity, Aβ1–40 and Aβ42 are neurotoxic because of modulation by γ-secretase. They are more likely to accumulate into oligomers, which are eventually deposited in areas such as the olfactory cortex, hippocampus, and other areas of the cortex to form amyloid plaques. Ultimately, Aβ1–40 and Aβ42 lead to synaptic dysfunction, neuronal death, and cognitive decline (Basisty et al., 2020).

### 2.2. Abnormal phosphorylation of tau protein

Tau protein is a soluble microtubule-associated protein that combines with other tubules to form microtubules that coordinate various cellular functions. Abnormal phosphorylated tau protein forms neurofibrillary tangles (NFTs) that are deposited in the cytoplasm, rendering it unable to perform normal biological functions such as maintaining microtubule stability, reducing dissociation, and inducing microtubule bunching (Jouanne et al., 2017). It is also closely associated with cognitive decline. Protein kinases and phosphatases regulate tau protein phosphorylation. Studies have shown that Aβ can affect the activity of glycogen synthase kinase 3β (GSK-3β) and other protein kinases and the stability of the PP system, thus inducing tau protein deposition (Hampel et al., 2019). A study of 107 participants using Tau positron emission tomography (PET) scans found that mild cognitive decline in precursor AD was mainly related to abnormal tau protein accumulation in the medial and infratemporal cortex (Jo et al., 2020).

### 2.3. Acetylcholine activity was decreased

Acetylcholine is a neurotransmitter closely related to cognitive functions in the brain, such as learning and memory. The severity of AD is positively correlated with the degree of cholinergic deficiency. Cholinergic deficiency in patients with AD affects the blood–brain barrier (BBB), reducing neuronal excitability and weakening memory and learning functions. The nucleus basalis of Meynert (NbM) is the cerebral cortex’s main source of cholinergic innervation. Extensive literature has shown that in the early stages of AD, patients have a significant loss of “large cell neurons” in the NbM and degeneration of nerve fibers (Ch4) in cholinergic NbM neurons. The degree of nerve fiber degeneration is associated with cognitive deficits (Cho et al., 2019). The number of Ch4 neurons is reduced by 80% in patients with AD compared with healthy controls (Babic Leko et al., 2021).

### 2.4. Glutamate toxicity

Glutamic acid is an excitatory amino acid in the nervous system that is involved in synaptic transmission, structural differentiation, learning, memory, and other neuronal functions. Ionotropic glutamate receptors (iGluRs) and metabotropic glutamate receptors (mGluRs) are distributed throughout the postsynaptic membranes of neurons. In patients with AD, the expression levels of vesicular glutamate transporters (VGLUT) 1 and 2 in the cerebral cortex are decreased, and the process of converting glutamic acid to glutamine is blocked, leading to excessive accumulation of glutamic acid between synapses. This accumulation acts on the anti-N-methyl-D-aspartate receptor (NMDAR), ultimately increasing Ca^2+^ concentration, neuroexcitatory toxicity, and neuronal apoptosis (Ogbodo et al., 2022).

### 2.5. Autophagy

Autophagy is a lysosome-mediated process that prevents abnormal protein aggregation and cell aging; it is essential for eliminating harmful substances in the body (Reddy and Oliver, 2019). Mitochondria play a crucial role in providing large amounts of adenosine triphosphate (ATP) for normal neuronal function. The removal of damaged mitochondria because of aging is vital for the maintenance of cellular homeostasis. Studies have shown that abnormal mitochondrial autophagy can lead to abnormal accumulation of the Aβ42 protein, even before the onset of the pathological symptoms of AD. Moreover, abnormal deposition of the Aβ42 protein has toxic effects on mitochondrial autophagy, inhibiting its normal function by suppressing key enzymes involved in mitochondrial metabolism (Luo et al., 2020).

### 2.6. Inflammatory response

Neuroinflammatory responses play a critical role in AD progression. Acute inflammation protects against brain injury and microglial cells act as phagocytes of the immune system. However, when the phagocytic capacity of microglia reaches maximum, their continuous activation results in the loss of their ability to clear Aβ plaques. Subsequently, the continuous deposition of Aβ plaques contributes to learning and memory dysfunction in patients with AD. Additionally, studies have shown that intestinal microbial disorders are closely associated with the occurrence of AD. Intestinal microbial disorders cause an increase in deoxycholic acid, which is deposited in the brain through the BBB, leading to apoptosis, reactive oxygen species (ROS) generation, inflammation, and neurodegeneration (Nho et al., 2019).

### 2.7. Mitochondrial hypothesis

The hypothesis of mitochondria-targeting drugs on AD included were as follows: (1) an improvement of the energy deficit related to neurodegeneration, including mitochondrial bioenergetics stimulants, mitochondrial biogenesis activators, and neuroprotectors and (2) increase in the resistance of mitochondria to the opening of mitochondrial permeability transition (MPT) pores (Shevtsova et al., 2021). There are some early signs in the early stage of AD and mild cognitive impairment (MCI), such as (1) the decrease in glucose consumption and disruption of mitochondrial bioenergetics (Chételat et al., 2003; Bachurin et al., 2018) and (2) disruption of glucose transport through BBB (Delbarba et al., 2016; Kuehn, 2020).

Alzheimer’s disease might be called “type 3 diabetes” because insulin resistance increases the risk of dementia (Kandimalla et al., 2017; Neth and Craft, 2017). Insulin is associated with the brain’s energy metabolism; insulin receptors are widely expressed in the brain’s temporal lobe and hippocampus, which control memory and language (Watson and Craft, 2003). Also, the insulin-sensitive glucose transporter GLUT4 is important for memory and cognitive functions, which is expressed in the brain area, particularly in the hippocampus. Finally, additional glucose supply contributes to the activation of brain bioenergetics.

The activity and expression of the mitochondrial respiratory chain (mRC) decreased in early AD and its animal model (Yao et al., 2009). An Aβ-induced mitochondrial dysfunction of AD model was made by a transgenic *Caenorhabditis elegans* strain, which expressed human Aβ peptide specifically in neurons (GRU102). It showed that alterations in the tricarboxylic acid (TCA) cycle metabolism; reduced activity of a rate-limiting TCA cycle enzyme, i.e., alpha-ketoglutarate dehydrogenase (α-KGD); and low-level Aβ expression in GRU102 result in increasing protein carbonyl content, specifically in mitochondria. Moreover, metformin (an anti-diabetes drug) recovered Aβ-induced metabolic defects, reduced protein aggregation, and normalized the lifespan of GRU102 (Teo et al., 2019). Furthermore, metformin decreased the blood glucose level, α-KGD activity, and formation of Àβ aggregates. It can even extend the life span of *C. elegans*, enhancing the mRC activity and mitochondrial fission (Wang et al., 2019).

### 2.8. “Multi-target” agents

Multiple pathogenic factors (e.g., Aβ, metal ions, metal-bound Aβ, and ROS) are found in the brain of patients with AD. One of the modern approaches for creating multitarget agents for AD treatment is polypharmacophore design—building hybrid molecules that are conjugates of two or more different pharmacophores linked together with spacers (Bolognesi and Cavalli, 2016; Han et al., 2018).

**FIGURE 1:**
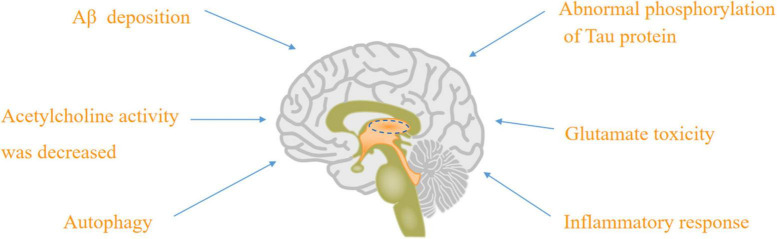
The physiological and pathological basis of AD: Aβ deposition, abnormal phosphorylation of tau protein, acetylcholine activity was decreased, glutamate toxicity, autophagy, and inflammatory response.

Alzheimer’s disease is a multifactorial neurodegenerative disease; therefore, logically multi-target drugs would be the best choice (Makhaeva et al., 2019). To date, there are five pharmacophores that demonstrate multi-target effects on AD, which are γ-carbolines, carbazoles, tetrahydrocarbazoles, phenothiazines, and aminoadamantanes. Biological activity of these compounds include inhibitory potency against AChE, butyrylcholinesterase (BChE), anti-carboxylesterase and anti-aggregation activities, and binding to the two sites of the NMDA subtype of the glutamate receptor to conduct potential cognition enhancement and neuroprotection against mitochondrial triggers of cell death (Makhaeva et al., 2019). There were selective BChE inhibitors (conjugates of γ-carbolines and phenothiazine I, γ-carbolines and carbazoles II, and aminoadamantanes and carbazoles III) as well as inhibitors of both cholinesterases (conjugates of γ-carbolines and methylene blue IV and bis-γ-carbolines with ditriazole-containing spacers V). These compounds exhibit combined potential for cognition enhancement, neuroprotection, and disease modification. Moreover, none of the conjugates exhibited high potency against carboxylesterase (CaE), thereby precluding potential drug–drug interactions through CaE inhibition (Makhaeva et al., 2019; Figure 1).

## 3. Therapeutic strategies for developing anti-AD drugs

The FDA has currently approved single-target drugs such as AChEI and NMDAR antagonists. There is a growing interest in developing multi-target drugs (Athar et al., 2021) that can address various aspects of AD pathology, including anti-Aβ deposition, tau protein phosphorylation, oxidative stress, and mitochondrial autophagy dysfunction. Many of these drugs are currently undergoing clinical trials.

### 3.1. Upcoming AD drugs targeting Aβ

The above FDA-approved therapies are only intended to ameliorate symptoms. Therefore, disease-modifying therapies are required to slow, modify, and control AD progression. The main mechanism of action of anti-Aβ drugs is to reduce the Aβ production, prevent its deposition, and accelerate its clearance.

#### 3.1.1. β-Secretase inhibitor

β-Secretase inhibitors primarily reduce the production of amyloid beta. However, clinical trials of inhibitors targeting β-site APP-cleaving enzyme 1 (BACE1, also known as β-secretase 1) have largely been unsuccessful. Verubecestat, Lanabecestat, and Atabecestat are some of the molecules from the acyl guanidine class that have successfully reached the later stages of clinical trials. Nevertheless, they all failed to reach the market because of toxicity or a lack of clinical efficacy (Patel et al., 2022).

Verubecestat (MK-8931) was the first compound to enter Phase III trials because of its ability to cross the BBB and improve bioavailability. However, two Phase III clinical trials, EPOCH and APECS, were terminated prematurely after the drug failed to improve cognitive decline in participants and increased adverse events (AE) (Doggrell, 2019; Egan et al., 2019; Moussa-Pacha et al., 2020; Patel et al., 2022).

Lanabecestat (AZD3293) was studied in two Phase II/III and Phase III trials: AMARANTH and DAYBREAK-ALZ. These trials were designed to demonstrate its ability to slow the progression of mild AD. However, it was not found to slow cognitive decline in patients with mild AD at the mid-stage of the trial. Therefore, the trial was terminated prematurely (Wessels et al., 2020).

Atabecestat (JNJ-54861911), a potent BACE1 inhibitor, reduces the Aβ production in treating AD. Two Phase I trials, NCT01978548 and NCT02360657, showed an average 67 and 90% reduction in Aβ1-40 in the cerebrospinal fluid (CSF) of patients with early AD who received daily doses of 10 and 50 mg atabecestat for 4 weeks (Timmers et al., 2018). However, in a Phase II/III randomized, double-blind, placebo-controlled study, the trial was stopped early because of serious liver-related AE, and cognitive deterioration was found to be reversible after a 6-month follow-up of patients with AD (Sperling et al., 2021).

The BACE-1 inhibitor, Umibecestat (CNP520), has high selectivity and brain penetration, and animal toxicology studies have shown that it has a sufficiently safe range without AEs such as hair loss, cardiovascular damage, or liver toxicity (Neumann et al., 2018). However, trials of umibecestat at doses of 15 and 50 mg were stopped in two clinical prevention studies after the participants showed deterioration in the Neuropsychological State Cognition Test, even displaying significant brain shrinkage and weight loss (Vormfelde et al., 2020).

Elenbecestat (E2609) is another BACE-1 inhibitor that is an aminothiazine derivative. It has been shown to reduce the Aβ level in CSF (Roberts et al., 2021). In preclinical studies, without evidence of hypopigmentation, Elenbecestat reduced Aβ protein levels in rat and guinea pig brains, CSF, and plasma (Moriyama et al., 2017; Hsiao et al., 2019). In an elenbecestat healthy volunteer Phase I study (E2609-A001-002), it showed that Aβ decreased at 50 mg and increased at 100 and 400 mg (ClinicalTrials.gov.NCT01511783). This result was supported by an elenbecestat Phase II study (E2609-G000-201), which showed that CSF Aβ decreased at 50 mg in patients with MCI and early mild AD. Unfortunately, two elenbecestat global Phase III studies (E2609-G000-301 or MissionAD1) (ClinicalTrials.gov.NCT02956486) and (E2609-G000-302 or MissionAD2) were terminated because of an unfavorable risk-benefit ratio (Miranda et al., 2021).

#### 3.1.2. γ-Secretase inhibitors

Semagacestat (LY450139) is a non-selective small-molecule γ-secretase inhibitor that targets the same mechanism as that of β-secretase inhibitors, aiming to reduce the deposition of Aβ amyloid protein. Two single-dose (140 mg), open-label, randomized crossover Phase III clinical trials showed that the clinical efficacy of semagacestat was independent of the preparation, food, and administration time. Additionally, the drug was well tolerated during the trial, and no safety concerns were reported (Willis et al., 2012). However, in a later Phase III trial (NCT00594568), the trial was terminated because of weight loss in patients treated with semagacestat and significantly higher rates of AEs, such as skin cancer and infection, than in the placebo group (Doody et al., 2013; Henley et al., 2014). Similarly, in a Phase II clinical trial of avagacestat in patients with mild-to-moderate AD, the trial was terminated because of the development of AEs such as brain microbleeds, diabetes, and skin cancer (Pinheiro and Faustino, 2019).

#### 3.1.3. Drugs that enhance Aβ clearance (immunotherapy)

The two main types of immunotherapeutic drugs that can enhance the immune clearance of pathogens are active immunity (achieved through vaccination) and passive immunity (achieved through the administration of monoclonal antibodies).

#### 3.1.4. Active immunity

The first anti-Aβ vaccine (AN1792) demonstrated the success of active immunotherapy in eliminating Aβ plaques, which could also be maintained for up to 14 years. However, in the Phase IIa clinical trial, approximately 6% of patients with AD treated with AN1792 developed meningoencephalitis (ME), leading to the termination of the trial. ME production may be related to the T-cell immune response (Nicoll et al., 2019).

A novel vaccine called ACC-001 was developed to avoid harmful T-cell responses and accelerate the clearance of Aβ plaques to address this issue (Pride et al., 2008). Phase II clinical trials of ACC-001 in patients with mild and moderate AD indicated that the vaccine had tolerable safety, regardless of whether the QS-21 adjuvant was used (Pasquier et al., 2016). Additionally, it was found that ACC-001 + QS-21 produced higher anti-Aβ antibody titers than the control group without QS-21 (Hull et al., 2017).

However, CAD106, an anti-Aβ vaccine containing peptide Aβ1-6, was terminated in another study of an AD prevention program because of abnormal changes in cognitive function, brain volume, and body weight in participants (Ohtake et al., 2017). In contrast, in Phase I clinical trials, ABvac40, the first active vaccine targeting the C-terminal of Aβ40, has shown good safety and tolerability (Lacosta et al., 2018).

#### 3.1.5. Passive immunity

Bapineuzumab is a humanized monoclonal antibody that specifically targets Aβ and aims to reduce the abnormal deposition of Aβ plaques. In a Phase II study, treatment-emergent adverse events (TEAEs), including agitation and urinary tract infections, were reported in patients with severe AD. Bapineuzumab also causes amyloid-related imaging abnormalities (ARIA) with effusion or edema (ARIA-E) and ferriflavin deposition (Salloway et al., 2018). However, in two Phase III trials (NCT00575055 and NCT00574132), bapineuzumab did not significantly improve cognitive function in patients with AD (Salloway et al., 2014).

In contrast, gantenerumab is an IgG monoclonal antibody that accelerates the clearance of Aβ plaques through Fc receptor-mediated phagocytosis. A PET substudy clinical trial showed that a 1,200 mg dose of gantenerumab could stably clear Aβ plaques (Klein et al., 2019). No serious adverse events were reported after large-volume subcutaneous injection of gantenerumab (Portron et al., 2020). This drug can potentially reverse the pathology of amyloid plaques significantly and may alter the course of the disease by slowing or stopping its clinical progression (Bateman et al., 2022).

Crenezumab (RO5490245) is an IgG4 antibody with a high affinity for Aβ plaque oligomers and can be administrated at higher doses. No serious adverse events were reported in the GP29523 or GP40201 studies (Dolton et al., 2021). Two other Phase III multicenter trials were halted during mid-stage reviews because of the lack of clinical effectiveness of crenezumab (Ostrowitzki et al., 2022).

Ponezumab (PF-04360365), an IgG2 monoclonal antibody, was well tolerated in the trial but did not significantly affect Aβ deposition (Landen et al., 2017). In a double-blind, placebo-controlled Phase III trial of solanezumab at a dose of 400 mg every 4 weeks in patients with mild AD, no significant improvement in cognitive decline was observed, and cognitive decline preceded dysfunction in patients with mild AD throughout the trial. These findings could provide new insights into the prevention and treatment of AD at an early stage (Honig et al., 2018; Liu-Seifert et al., 2018).

Donanemab is currently in Phase III trials for the treatment of early AD. In the four Donanemab studies, 228 participants receiving Donanemab and 168 participants receiving placebo had low baseline levels of complete amyloid clearance. It was also found that in the Donanemab group, Tau accumulation was slower, and Donanemab was associated with ARIA-related AEs during the trial (Mintun et al., 2021; Rashad et al., 2022; Shcherbinin et al., 2022).

**FIGURE 2:**
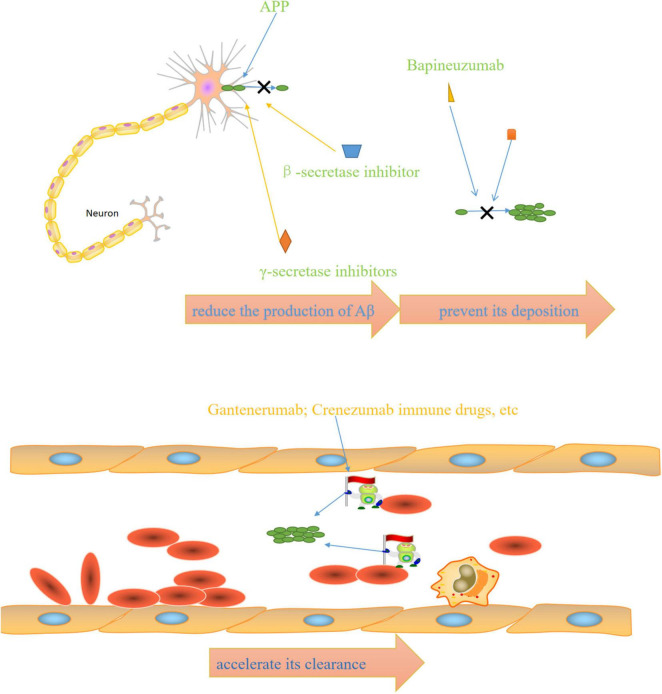
Mechanisms of anti-AD drugs: β-secretase inhibitors and γ-secretase inhibitors reduce the production of Aβ, bapineuzumab and other drugs prevent its deposition, immune drugs such as gantenerumab and crenezumab accelerate its clearance.

Aducanumab, a monoclonal antibody that targets soluble and insoluble Aβ aggregates (IgG1) and selectively binds to Aβ, received accelerated approval from the US FDA on 7 June 2021. It was the first new drug for the treatment of AD for 20 years, following the approval of the US FDA. It is currently under regulatory review in Japan and Europe to assess its safety and tolerability for long-term use (Dhillon, 2021). However, this drug can significantly increase the incidence of ARIA (Cummings et al., 2021; Table 1 and Figure 2).

**TABLE 1:** The anti-Aβ drugs are currently undergoing clinical trials or just approved including name of drugs, mechanism, company, and clinical trials.

Name of drug	Mechanism	Company	Clinical status
Verubecestat (MK-8931)	BACE 1 inhibitor	Merck Sharp (USA)	Phase III (terminated in 2019)
Lanabecestat (LY3314814)		Eli Lilly (USA)	Phase III (terminated in 2018)
Atabecestat (JNJ-54861911)		Janssen (USA)	Phase IIb/III (terminated in 2018)
Umibecestat (CNP520)		Novartis, Amgen, and Banner (USA)	Phase II/III (terminated in 2019)
Elenbecestat (E2609)		Biogen and Eisai (USA)	Phase III (terminated in 2019)
Semagacestat	γ-Secretase inhibitors	Eli Lilly (USA)	Phase III (terminated in 2011)
Avagacestat		Bristol-Myers Squibb (USA)	Phase II (terminated in 2013)
ACC-001	Active immunity	JANSSEN (USA)	Phase II (completed)
CAD106		Novartis (USA)	Phase II (terminated in 2010)
ABvac40		Araclon Biotech S.L.	Phase I (completed)
Bapineuzumab	Passive immunity	JANSSEN, Pfizer (USA)	Phase III (completed)
Gantenerumab		Hoffmann-La Roche	Phase III (completed)
Crenezumab		Hoffmann-La Roche	Phase III (terminated in 2019)
Ponezumab		Pfizer (USA)	Phase I (completed)
Solanezumab		Eli Lilly (USA)	Phase III (terminated in 2017)
Donanemab		Eli Lilly (USA)	Phase III (ongoing)
Aducanumab		Biogen	Approved (7th June 2021)

### 3.2. Anti-tau drugs

The role of the tau protein is not fully understood, but studies have shown that it plays an important role in the assembly and stabilization of cytoskeletal microtubules. Abnormal hyperphosphorylation of Tau (p-tau) reduces its affinity with bound microtubules, and Tau’s abnormal phosphorylation leads to the aggregation and formation of NFT. The treatment of anti-tau drugs mainly includes three aspects: preventing tau hyperphosphorylation and aggregation, stabilizing microtubules, and accelerating tau clearance.

#### 3.2.1. GSK-3β inhibitor

Tau phosphorylation is regulated by protein kinase and phosphatase. Among these, GSA-3β is associated with p-tau production and subsequent neuronal degeneration in AD (Wegmann et al., 2021). GSK-3β inhibitors can prevent tau hyperphosphorylation. Studies have shown that GSK-3β can reduce abnormal Tau phosphorylation and amyloid protein production *in vitro* and *in vivo*, a promising disease-modifying therapy for AD. Tideglusib, a thiadiazolone that irreversibly inhibits GSK-3β and reduces tau phosphorylation, did not show any clinical benefit in a double-blind, placebo-controlled Phase II trial demonstrating the clinical efficacy of GSK-3 inhibitors in AD and is subject to further study (Lovestone et al., 2015). Lithium was first used in psychiatry and was discovered by Australian psychiatrist John Cade in 1949 and has been widely used to treat manic episodes. In recent years, Lithium has been found to be an inhibitor of GSK3, involved in glucose metabolism, cell signaling and proliferation, and glial cell function regulation. Lithium can prevent amyloid formation and Tau hyperphosphorylation. There have been some case reports as well as case control studies showing that Lithium can reduce the symptoms of AD. However, clinically available Lithium has serious side effects (SAE) with long-term use. It requires constant monitoring of Lithium concentrations in the blood; safer and more effective Lithium is needed for clinical use (Haussmann et al., 2021; Hu et al., 2022; Muronaga et al., 2022; Luca and Luca, 2023).

#### 3.2.2. Tau aggregation inhibitor

Tau accumulation is associated with neuron loss. Tau aggregation inhibitors such as methylthioninium chloride (methblue) and hydromethanesulfonate (LMTM) can reduce Tau accumulation.

Methylthioninium chloride (methylene blue) is also a drug with a long history of use, primarily in malaria, methemogenemia, and carbon monoxide poisoning, as well as histological dyes. Methylthioninium chloride failed to show clinical benefit for AD in a 24-week Phase II study (Tucker et al., 2018). LMTM is a compound with a higher bioavailability and lower toxicity than methylthioninium chloride. In one Phase III trial involving mild to moderate AD, LMTM failed to slow cognitive or functional decline, and another phase III trial involving healthy older people with mild to moderate AD is still being conducted (Seripa et al., 2016; Hashweh et al., 2020).

TRx-0014 (Rember) was completed in a Phase II study of patients with mild to moderate AD.

It showed improvements in the Alzheimer’s Disease Assessment Scale–Cognitive Subscale (ADAS-Cog) over 24 weeks, as well as in the Alzheimer’s Disease Cooperative Study–Clinical Global Impression of Change scale (ADCS-CGIC), the MMSE, and cerebral blood flow assessed by HMPAO-SPECT. Unfortunately, TRx-0014 showed no statistically significant effect on cognition in patients with mild AD (NCT00515333) (Wischik et al., 2015).

A Phase II trial of TRx0237 (LMT/hydromethylthionine) in mild-moderate AD (NCT01626391) was terminated early for administrative reasons (ClinicalTrials.gov.NCT01626391). Moreover, two Phase III studies [(NCT01689246)/the European Union Clinical Trials Registry (2012-002866-11), (NCT01689233) and the European Union Clinical Trials Registry (21012-002847-28)] confirmed that TRx0237 improved the cognition in patients with mild to moderate AD, such as changes in ADAS-Cog and Alzheimer’s Disease Cooperative Study– Activities of Daily Living Inventory (ADCS-ADL) (Gauthier et al., 2016; Wilcock et al., 2018). A Phase II/III trial of TRx0237 Monotherapy in participants with AD (LUCIDITY/NCT03446001) was completed recently. The use of TRx0237 reflected as improvements in ADAS-Cog11 and ADCS-ADL23 (ClinicalTrials.gov.NCT03446001).

#### 3.2.3. Stable microtubules

Davunetide (alternative names: NAP or NAPVSIPQ or A-L108 or CP201) is an eight amino-acid peptide derived from the neuroprotective fragment of activity-dependent neuroprotective protein (ADNP) (Gozes et al., 2009). Davunetide is a Src homology 3 (SH3) domain-ligand association site responsible for controlling signaling pathways regulating the cytoskeleton, direct microtubule end-binding protein interaction facilitating microtubule dynamics, and Tau microtubule interaction at the microtubule end-binding protein site EB1 and EB3 (Gozes and Shazman, 2023). Davunetide may contribute to the progression of several CNS disorders, such as Autism, Schizophrenia, AD (Idan-Feldman et al., 2011; Sragovich et al., 2017, 2019), and Progressive Supranuclear Palsy (Dale et al., 2020; VandeVrede et al., 2020). A placebo-controlled, ascending-dose 12 weeks Phase I study in participants with amnestic MCI (AL-108-21) was completed in 2013, showing that davunetide was generally safe and well tolerated. However, it failed to detect a statistically significant difference between the treatment groups on the composite cognitive memory score of efficacy data (Morimoto et al., 2013).

Epothilones are derived from *Sorangium cellulosum* and inhibit tubulin depolymerization, thus leading to the death of cancer cells (Ye et al., 2023). Moreover, Epothilone D can bind to tau protein, thus effectively preventing nerve injury and improving cognitive performance in mouse models of AD (Brunden et al., 2010; Guo et al., 2020). Epothilone D (KOS-862) showed manageable toxicity, favorable PK profile, and the suggestion of clinical activity in Phase I clinical study of patients with advanced solid tumors and lymphoma (Konner et al., 2012) and in a Phase III trial in patients with advanced or metastatic breast cancer (Vahdat, 2008). Unfortunately, there were no publications regarding clinical trials of Epothilone D therapy in AD.

#### 3.2.4. Active immunity

AADvac1 is a peptide that contains one of the epitopes of antibody DC8E8 (294KDNIKHVPGGGS305). AADvac1 is conjugated to keyhole limpet hemocyanin (KLH) along with aluminum hydroxide as an adjuvant. AADvac-1 therapy in patients with mild-to-moderate AD was completed in Phase I trials of (NCT01850238), without aberrant immune response or microhemorrhages (Novak et al., 2017, 2019); similar results were found in a follow-up study of 72 weeks (NCT02031198) (Novak et al., 2018). Moreover, cognitive decline (ADAS-cog11 value) in patients with mild-to-moderate AD was significantly reduced by AADvac1 (Novak et al., 2018). These results were confirmed in an AADvac-1 Phase II clinical trial (NCT02579252) (ClinicalTrials.gov.NCT02579252).

ACI-35 is a liposomal-anchored 16-amino acid tetra-palmitoylated phospho-tau peptide (393VYKSPVVSGDTSPRHL408) (Theunis et al., 2013). ACI-35 decreased soluble and insoluble Tau in tau-transgenic mouse models (Theunis et al., 2013).

#### 3.2.5. Passive immunity

Gosuranemab is a humanized mouse monoclonal antibody (IPN002), which recognizes a phosphorylated epitope in the N-terminal region of Tau consisting of amino acid residues 15AGTYGLGDRK24 and targets extracellular Tau (Qureshi et al., 2018). Gosuranemab was found to be safe and well-tolerated in Phase 1 trials (NCT02460094) conducted on patients with PSP. Additionally, it demonstrated a reduction in unbound N-terminal Tau in CSF (Boxer et al., 2019). Unfortunately, AD biomarkers such as total Tau and ptau181 were not reduced by Gosuranemab (Tatebe et al., 2017; Yang et al., 2018). A Phase II clinical trial of Gosuranemab (TANGO trial, NCT03352557) is ongoing, with a completion date of 2024.

Tilavonemab (ABBV-8E12/C2N8E12/HJ8.5) is the humanized anti-Tau IgG4 antibody, which was found to be safe and tolerable as IV injections in Phase I trials (NCT02494024) (West et al., 2017). A Phase II trial of Tilavonemab for early AD (NCT02880956) found that Tilavonemab was tolerated generally well but found non-significant efficacy in treating patients with early AD (Florian et al., 2023).

Zagotenemab (LY3303560, MC-1 IgG1) is a humanized antibody that recognizes a conformational Tau epitope with a primary epitope located in the N-terminal region (Alam et al., 2017). Two Phase I trials of zagotenemab in healthy volunteers and patients with mild to moderate AD (NCT02754830 and NCT03019536) have been completed. However, no reports were released for unknown reasons (ClinicalTrials.gov.NCT02754830 and ClinicalTrials.gov.NCT03019536). Recently, a Phase II trial of zagotenemab (NCT03518073) was completed, showing that zagotenemab improves the clinical characteristics of patients with early AD. However, the trial showed an SAE occurrence of approximately 17% (ClinicalTrials.gov.NCT03518073).

Semorinemab (RO7105705) targets maximum binding across different extracellular Tau species, which was confirmed by preclinical studies in mouse models (Lee et al., 2016). Phase 1 Semorinemab (NCT02820896) studies have been completed; however, the corresponding report has not been made available. The study details can be found at https://beta.clinicaltrials.gov/study/NCT02820896. Recently, two ongoing Phase II trials have been completed: one involving participants with prodromal/probable AD (TAURIEL trial, NCT03289143) and another involving participants with moderate AD (NCT03828747). Both trials showed improvement in the clinical characteristics of patients with AD (Teng et al., 2022; ClinicalTrials.gov.NCT03828747; Table 2).

**TABLE 2:** The anti-tau drugs are currently undergoing clinical trials including name of drugs, mechanism, company, and clinical trials.

Name of drug	Mechanism	Company	Clinical status
Tideglusib	GSK-3β inhibitor	Noscira SA	Phase II (completed)
Lithium		National Institutes of Health Clinical Center (USA)	Phase II (completed)
Methylthioninium chloride (methylene blue)	Tau aggregation inhibitor	Allon Therapeutics/ Bristol-Myers/ Squibb (USA)	Phase II (completed) and Phase III (ongoing)
TRx-0014 (Rember)		University of Aberdeen (UK)	Phase II (completed)
TRx-0237 (LMT/hydromethy lthionine)		TauRx	Phase II/III (completed)
Hydromethanesul fonate (LMTM)		The University of Texas Health Science Center at San Antonio (USA)	Phase II (ongoing)
Davunetide (NAP)	Stable microtubules	Allon Therapeutics Inc.	Phase I (completed)
AADvac1	Active immunity	Axon Neuroscience/ Weil am Rhein	Phase I/II (completed)
ACI-35		AC Immune	Phase I (completed)
Gosuranemab (BIIB092, BMS-986168, IPN007/IPN002) Tilavonemab (ABBV-8E12/ C2N8E12/HJ8.5)	Passive immunity	iPerian/Bristol-Meyers Squibb/Biogen (USA) C2N Diagnostics/ AbbVie	Phase II (ongoing) Phase II (ongoing)
Zagotenemab (LY3303560, MC-1 IgG1)		Eli Lilly (USA)	Phase II (ongoing)
Semorinemab		AC Immune/ Genentech/ Hoffmann-La Roch	

### 3.3. Mitochondria-targeting drugs on AD

#### 3.3.1. Insulin

Phase II/III clinical trials on insulin for AD (NCT01767909) have been completed. However, no cognitive or functional benefits were observed with a 12-months period of intranasal insulin treatment, although no clinically important AE was associated with the treatment (Craft et al., 2020).

#### 3.3.2. Mitochondrial enhancers

“Mitochondrial enhancers” therapy in the early stages of AD included coenzyme Q (CoQ) and its synthetic analog, idebenone, which stimulate the mitochondrial electron transport chain activity, increase ATP production, and exhibit antioxidant- and free-radical-scavenging activity. Studies showed that the oxidized/total CoQ ratio was increased in the CSF of patients with AD (Isobe et al., 2009; Orsucci et al., 2011). Similar results were also observed in animal models, including older animals and diabetic rats (Yang et al., 2016). Clinical trials on these drugs (NCT00117403) have been completed, and they failed to show statistically significant efficacy (Gutzmann et al., 2002; Thal et al., 2003; Salloway et al., 2021).

Another mitochondrial enhancer, methylene blue, is a member of the phenothiazines family, which interacts with mitochondria and induces an alternative electron transfer to cytochrome oxidase, thus increasing its activity and possessing antioxidant properties (Tucker et al., 2018). Methylene blue is also a multi-target drug on several biotargets, such as mitochondria, membrane-associated transporters, and ion channels (Saitow and Nakaoka, 1997) and the activity of cholinergic, monoaminergic, or glutamatergic synaptic neurotransmission (Ramsay et al., 2007; Vutskits et al., 2008; Shevtsova et al., 2021). Unfortunately, a compound of methylene blue [Leuco-methylthioninium bis (hydromethanesulfonate; LMTM)] has failed to show a statistically significant positive effect in phase III clinical trials (NCT01689246) on AD (Gauthier et al., 2016). However, another phase III clinical trial showed that LMTM improved cognitive function, brain atrophy, and blood glucose in patients with AD (Wilcock et al., 2018). Thus, further evidence is needed to support its efficacy.

Mitochondrial dysfunction can be recovered through mitochondrial biogenesis, which includes peroxisome proliferator-activated receptor (PPAR) and transcription coactivators such as PPARγ coactivator-1 (PGC-1) family, nuclear transcription factors including nuclear respiratory factors 1 (NRF-1) and 2 (NRF-2), and the mitochondrial transcription factor (ÒFAM). The impairment of PGC-1α-mediated mitochondrial biogenesis appears in patients with AD (Qin et al., 2009) and AD model-3xTg mouse (Singulani et al., 2020).

Peroxisome proliferator-activated receptor (α, β/δ, γ) agonist, bezafibrate, decreases the tau protein level and microglia activation, enhances mitochondrial biogenesis, and improves behavioral characteristics in P301s transgenic mice (Dumont et al., 2012). Unfortunately, there were no clinical trial reports on Bezafibrate for AD treatment in ClinicalTrials.gov.

In mouse models, other PPAR-γ agonists such as thiazolidinediones, pioglitazone, and rosiglitazone showed improvement in memory. Rosiglitazone improved cognitive functions only in a small group of patients with MCI. However, some extensive clinical trials (REFLECT-2 and REFLECT-3) did not show a statistically significant efficacy (Harrington et al., 2011; Shevtsova et al., 2021). Recently, multiple trials on rosiglitazone therapy for AD, including a Phase IIb (NCT00334568) and Phase III [(AVA105640; NCT00428090), (AVA102677; NCT00550420); (study AVA10267, NCT00348309); (study AVA102670; NCT00348140)] trials were reported. These reports showed that six protein-predictive biomarkers (IL6, IL10, CRP, TNF, FABP-3, and PPY) could accurately classify 100% of rosiglitazone treatment responders. Unfortunately, this report did not mention any improvement in the cognitive function of patients with AD (O’Bryant et al., 2021).

#### 3.3.3. Mitochondrial permeability transition inhibitors

Mitochondrial permeability transition inhibitors can prevent neurodegenerative processes and can be considered potential neuroprotectors. MPT inhibitors include modulators of mitochondrial calcium homeostasis and antioxidants. The MPT pore is considered a complex consisting of poly-R-3-hydroxybutyrate, polyphosphates, and calcium cations (PHB/polyp/Ca^2+^ complex) (Pavlov et al., 2005). An experiment showed that a decrease in the polyphosphate level increases the calcium retention capacity of mitochondria and reduces the probability of Ñà^2+^-induced MPT pore opening (Abramov et al., 2007).

Cyclophilin D (peptidyl-prolyl cis–trans isomerase, PPIase) might be a master regulator of mitochondrial function, such as the mitochondrial redox status, presence of inorganic phosphate, and state of respiratory chain components, including complex I of the MRC, creatine kinase, and translocator protein (TSPO) peripheral benzodiazepine receptor (Azarashvili et al., 2015; Bernardi et al., 2015; Gutiérrez-Aguilar and Baines, 2015; Porter and Beutner, 2018). Cyclophilin family members have co-operations. For example, cyclophilin D is regulated by cyclosporin A through calcium. Cyclophilin D interacts with cardiolipin to release cytochrome C from mitochondria via tau protein-441, α-synuclein, and β-amyloid oligomers (Camilleri et al., 2013). There were some specific inhibitors, including cyclosporin À, alisporivir (Debio025) (Schiavone et al., 2017), N-methyl-4-isoleucine-cyclosporin (NIM811) (Springer et al., 2018), low-molecular-weight cyclophilin D ligands (4-aminobenzenesulfonamide derivative C-9) (Valasani et al., 2014, 2016), and cyclophilin D-independent MPT inhibitors (imidazole, thiadiazole, urea derivatives, N-phenylbenzamides, cinnamic anilides, and isoxazoles) (Valasani et al., 2016). Unfortunately, there are no clinical trials or animal model reports regarding these compounds (Shevtsova et al., 2021).

Dimebon prevents the opening of MPT pores and is considered a treatment for AD. Dimebon showed strong neuroprotective and cognition-enhancing effects in different animal models (Bachurin et al., 2001). Phase II clinical trials (NCT00377715) showed that Dimebon had a strong beneficial effect on memory and cognition in patients with AD (Doody et al., 2008). Unfortunately, this result was not supported by a phase III trial conducted in multiple centers, possibly because of the involvement of a heterogeneous population with various neuropathologies unrelated to AD symptoms (MacKay et al., 2010).

Melatonin and its precursor, N-acetylserotonin (NAS), not only have the receptor-defined hormonal effect but also act as antioxidants and are accumulated in mitochondria. They stimulate the MRC, inhibit the MPT, and possess significant neuroprotective potential (Pandi-Perumal et al., 2013; Zhou et al., 2014; Tarocco et al., 2019; Shevtsova et al., 2021). Melatonin and NAS affect MPT induction conditions and have multimodal capabilities, such as regulators of endogenous and local MPT and synaptic and neuronal viability (Tarocco et al., 2019). One clinical trial in Japan showed that melatonin significantly prolonged sleep time (Asayama et al., 2003). Unfortunately, there was no significant improvement in sleep or agitation in two different clinical trials on melatonin treatment for AD in the USA (Singer et al., 2003; Gehrman et al., 2009). Interestingly, a significant improvement in cognitive performance, such as IADL and MMSE, was observed in a 24-weeks clinical trial on prolonged-release melatonin (PRM, also called Piromelatine) therapy for patients with AD, particularly in those with insomnia comorbidity (Wade et al., 2014). However, a recently completed phase II clinical trial (ReCognition, NCT02615002) on Piromelatine therapy for AD showed no statistically significant improvement in cognitive functions (Schneider et al., 2022).

Translocator protein density has been used as a biomarker for neuroinflammation in AD (Chen and Guilarte, 2008). Recently it showed that TSPO binding was greater in patients with AD than in age-matched controls or patients with MCI who had a positive amyloid scan (Kreisl et al., 2013). It showed that increased TSPO binding might play a pathophysiological role in the transition from MCI to AD (Lyoo et al., 2015). In a clinical trial (NCT00613119), TSPO binding (*V*T/*f*P)1) was greater in patients with AD than in healthy controls in expected temporoparietal regions and 2) was not significantly different among the three groups in the cerebellum (Lyoo et al., 2015; Table 3).

**TABLE 3:** Mitochondria-targeting drugs are currently undergoing clinical trials including name of drugs, mechanism, company, and clinical trials.

Name of drug	Mechanism	Company	Clinical status
Insulin (Humulin-RU-100)	Reduce blood glucose	Lilly (USA)	Phase II/III (completed)
Idebenone	mitochondrial enhancers	Wilhelm Griesinger Hospital (Germany)	Phase III (completed)
coenzyme Q	Antioxidants	National Institute on Aging (NIA), Alzheimer’s Disease Cooperative Study (ADCS), Takeda America	Phase I/II (completed)
hydromethanesul fonate; (LMTM)		TauRx Therapeutics	Phase III (completed)
Rosiglitazone	PPAR-γ agonists	GlaxoSmithKline	Phase II (completed)
Melatonin	MPT inhibitors	Oregon Health and Science University	Phase II (completed)
prolonged-release melatonin (Circadin), Piromelatine		Neurim Pharmaceuticals Ltd. (Israel)	Phase II (completed)

### 3.4. “Multi-target” agents on AD

#### 3.4.1. γ-Carbolines

Dimebon is a compound of γ-carboline derivatives that conjugates with methylene blue (Bachurin et al., 2019; Makhaeva et al., 2019). Dimebon is a multitarget agent; its activities include protecting neurons from death, reducing the development of proteopathy, and increasing autophagy (Shevtsova et al., 2014; Skvortsova et al., 2018; Ustyugov et al., 2018). However, owing to a lack of statistically significant efficacy, the use of dimebon for AD was not confirmed through a phase II clinical trial (NCT00377715) (Doody et al., 2008).

#### 3.4.2. Phenothiazine

Phenothiazine does not inhibit AChE and has a rather low anti-BChE activity, which was supported by molecular docking (Makhaeva et al., 2015). In a double transgenic mouse AD model, phenothiazine-based theranostic compounds inhibited Aβ aggregation and might act as imaging probes for amyloid plaques in AD on near-infrared fluorescent (NIRF) imaging (Dao et al., 2017). Unfortunately, there is no clinical trial report currently available on phenothiazine therapy for AD in the PubMed database.

#### 3.4.3. Carbazoles

P7C3 is a neuroprotective aminopropyl carbazole identified on studies of postnatal hippocampal neurogenesis (Voorhees et al., 2018). P7C3 was named as it is the third compound (C3) of the seventh pool (P7) and has protective action on young hippocampal neurons in preventing neuron death; it has also been shown to inhibit cognitive decline in terminally aging rats (Pieper et al., 2010). Moreover, P7C3 molecules enhance the flux of nicotinamide adenine dinucleotide (NAD) in mammalian cells (Wang et al., 2014) and indirectly inhibit other critical cell death signaling events (Gu et al., 2017). P7C3 treatment shows neuroprotective effect in different animal models, such as amyotrophic lateral sclerosis (Tesla et al., 2012), Parkinson’s disease (De Jesús-Cortés et al., 2012, 2015; Naidoo et al., 2014; Gu et al., 2017), traumatic brain injury (Dutca et al., 2014; Yin et al., 2014; Vázquez-Rosa et al., 2020), psychological stress–related hippocampal cell death (Walker et al., 2015), peripheral nerve crush injury (Kemp et al., 2015), stroke (Kemp et al., 2015), and AD (TgF344-AD rat model) (Cohen et al., 2013; Voorhees et al., 2018). Unfortunately, there is no clinical trial report available on P7C3 therapy for AD in the PubMed database.

#### 3.4.4. 5-HT

Idalopirdine is a novel selective 5-HT6 receptor antagonist that binds with ChEI, potentiates central acetyl choline levels and neuronal activity, and improves cognition in animal models (Herrik et al., 2016; Amat-Foraster et al., 2017).

A phase II, proof-of-concept (PoC) study of idalopirdine plus donepezil therapy for AD showed a significant improvement in cognitive performance of AD, such as in ADAS-cog and MMSE scores (Wilkinson et al., 2014). However, phase III development programs for idalopirdine therapy (“OLEX,” idalopirdine only and “MEMOLEX,” idalopirdine plus memantine) for AD showed no statistically significant efficacy.

AVN-101 is a very potent 5-HT7 receptor antagonist that blocks 5-HT6, 5-HT2A, and 5HT-2C receptors as well as histamine H1 and adrenergic 2A, 2B, and 2C receptors. AVN-101 shows good oral bioavailability, facilitates BBB permeability, and has a low toxicity and reasonable efficacy in animal models of CNS diseases (Ivachtchenko et al., 2016). Moreover, a phase I clinical study indicated that the AVN-101 is well tolerated (Ivachtchenko et al., 2016).

#### 3.4.5. Tyrosine kinase inhibitor

Masitinib is an oral tyrosine kinase inhibitor that has demonstrated neuroprotective action in neurodegenerative diseases via inhibition of mast cell and microglia/macrophage activity, such as in cases of multiple sclerosis (AB07002) (Vermersch et al., 2022). Recently, a phase III clinical trial of masitinib therapy for AD (AB09004, NCT01872598) was completed, which demonstrated that masitinib causes significant improvement in ADAS-cog and ADCS-ADL scores (Dubois et al., 2023; Table 4).

**TABLE 4:** Multi-targets drugs are currently undergoing clinical trials including name of drugs, mechanism, company, and clinical trials.

Name of drug	Mechanism	Company	Clinical status
Dimebon (Latrepirdine)	Multi-targets	Medivation (USA), Pfizer, Novokuznetsk (Russia)	Phase III (completed)
Masitinib	Tyrosine kinase inhibitor	AB Science (France)	Unknown
Idalopirdine	5-HT6 receptor antagonist	H. Lundbeck A/S (Denmark)	Phase II/III (completed)
AVN-101		Avineuro Pharmaceuticals Inc. (USA)	Phase I (completed)

## 4. Discussion

Alzheimer’s disease is a neurodegenerative disease with increasing annual incidence. However, the pathogenesis of AD is complex, and its etiology has not been fully elucidated. Research and development of therapeutic drugs for AD are still in progress. Since AD has an insidious onset and slow disease progression, it can take up to 20 years from the onset of pathological changes to the appearance of clinically significant symptoms. Therefore, the early treatment of AD is crucial in controlling its progression. Anti-Aβ amyloid drugs are currently the focus of clinical trials; however, most clinical trials have been terminated because of AE and poor efficacy. For example, a clinical trial on aducanumab, donanemab, lecanemab, and other anti-Aβ drugs concluded that Aβ plaque clearance was closely related to the occurrence of ARIA-E (Wang et al., 2022). The high incidence of ARIAs suggests a need to clarify the early benefits of such interventions when conducting clinical trials (Loureiro et al., 2020). In clinical studies on donanemab, a trend toward slower Tau accumulation was observed. Thus, a future research direction would be to explore the relationship between reduced Aβ plaques and Tau levels, to achieve meaningful benefits for patients with AD. The accelerated FDA approval of aducanumab brought hope for AD drug development, and we look forward to more effective and economical treatments for patients with AD.

## 5. Conclusion

In conclusion, although there has not been a curative breakthrough in drug therapy for AD, progress is being made; new drugs with good efficacy, few adverse reactions, and economic feasibility will certainly be developed in the near future.

## Author contributions

YP received funding support and developed the research hypothesis. YP, HJ, Y-hX, QC, S-yY, M-qD, and SL wrote the main manuscript. All authors jointly wrote the final manuscript as the end product.
